# Resveratrol and Ophthalmic Diseases

**DOI:** 10.3390/nu8040200

**Published:** 2016-04-05

**Authors:** Khaled K. Abu-Amero, Altaf A. Kondkar, Kakarla V. Chalam

**Affiliations:** 1 Department of Ophthalmology, University of Florida College of Medicine, FL 32209, USA; abuamero@gmail.com; 2 Glaucoma Research Chair, Department of Ophthalmology, College of Medicine, King Saud University, Riyadh 11424, Saudi Arabia; akondkar@gmail.com

**Keywords:** age-related macular degeneration, cataract, diabetic retinopathy, eye, glaucoma, ocular disease, phytochemicals

## Abstract

Resveratrol, a naturally occurring plant polyphenol found in grapes, is the principal biologically active component in red wine. Clinical studies have shown that resveratrol due to its potent anti-oxidant and anti-inflammatory properties are cardio-protective, chemotherapeutic, neuroprotective, and display anti-aging effects. Oxidative stress and inflammation play a critical role in the initiation and progression of age-related ocular diseases (glaucoma, cataract, diabetic retinopathy and macular degeneration) that lead to progressive loss of vision and blindness. *In vitro* and *in vivo* (animal model) experimental studies performed so far have provided evidence for the biological effects of resveratrol on numerous pathways including oxidative stress, inflammation, mitochondrial dysfunction, apoptosis, pro-survival or angiogenesis that are implicated in the pathogenesis of these age-related ocular disorders. In this review, we provide a brief overview of current scientific literature on resveratrol, its plausible mechanism(s) of action, its potential use and current limitations as a nutritional therapeutic intervention in the eye and its related disorders.

## 1. Introduction

Oxidative stress and inflammation play critical roles in the initiation and progression of age-related ocular diseases, including glaucoma, cataract, diabetic retinopathy, and macular degeneration that lead to progressive loss of vision and blindness if untreated [1,2,3]. There is accumulating evidence to suggest that phytochemicals, with their antioxidant and anti-inflammatory properties, may have a potential role in the prevention and treatment of these age-related ocular disorders [4]. Among the several other phytochemicals there is an increasing interest in the therapeutic effects of resveratrol on the eye, particularly in terms of disease prevention.

Resveratrol (3,5,4′-trihydroxy-*trans*-stilbene) is a stilbenoid, a type of natural phenol, and a phytoalexin produced naturally by several plants in response to injury or when the plant is under attack by pathogens, such as bacteria or fungi [5]. Food sources of resveratrol include the skin of grapes, blueberries, raspberries, and mulberries. Interest in resveratrol began with the “French Paradox” describing the cardiovascular health benefits of red wine, which contains high concentrations of non-flavonoid resveratrol, to explain a comparatively low incidence of cardiovascular events among the French populations despite a high dietary consumption of saturated fat [6]. Since then, more research has focused on the biological effects of resveratrol, showing that resveratrol plausibly due to its potent anti-oxidative and anti-inflammatory properties can be cardioprotective [7], neuroprotective [8], chemotherapeutic [9], and exhibit anti-aging effects [10].

This review provides a brief overview of current scientific literature on resveratrol, its plausible mechanism(s) of action, its potential use and current limitations as a nutritional therapeutic intervention in the eye and its related disorders. A literature search was performed using PubMed, Web of Knowledge and Google for articles published up to 2015. Specific keywords, such as resveratrol, eye, eye disease, ocular, ocular disease, glaucoma, cataract, macular degeneration, diabetes, anti-oxidant, anti-inflammatory were used in combination(s) to filter the searches. Relevant information pertaining to the focus of this review was included.

## 2. Sources

### 2.1. Food Sources

Resveratrol is a polyphenol that is found in plants. Grapes, red wine, grape juice, peanuts, cocoa, and berries of *Vaccinum* species, including blueberries, bilberries, and cranberries are particularly rich sources [11]. Although red wine is a relatively rich source of resveratrol, other polyphenols are present in red wine at considerably higher concentrations than resveratrol, which is only a minor compound in the complete set of grape and wine polyphenols [12]. A myriad of natural and synthetic analogues of resveratrol and their isomer, adducts, derivatives and conjugates are known [13]. These compounds differ in structure type, number and position of substituents (e.g., hydroxyl, methoxyl, halogenated, glycosylated, esterified), presence or absence of stilbenic double bonds, modified steroisomery and oxidative dimerization to form oligomers and have distinct biological properties [13,14]. Resveratrol has a stilbene structure, consisting of two aromatic rings connected by a methylene bridge. There exist two structurally distinct forms of resveratrol, namely *cis*- and *trans*-resveratrol. The predominant form of resveratrol in grapes and grape juice is *trans*-resveratrol-3-*O*-β-glucoside (*trans*-piceid). Many wines also contain significant amounts of *cis*-resveratrol [12]. Estimates of resveratrol content of some beverages and foods are listed in Table 1 [11].

### 2.2. Supplements

Resveratrol from root extracts of *Polygonum cuspidatum* (also known as *Fallopia japonica*, Japanese knotweed), red wine and grape extracts (from *Vitis vinifera*) are readily available on the market as dietary supplements. These supplements may contain anywhere from less than 1 milligram (mg) to 500 mg of resveratrol per tablet or capsule, but its safety and efficacy in the prevention of chronic disease in humans are unknown.

## 3. Metabolism and Bioavailability

Pharmacokinetic studies of *trans*-resveratrol in humans found that the serum level of unmetabolized resveratrol is very low after oral or intraperitoneal administration of the drug. Recently, Beaudeux and colleagues have published an excellent review providing insights into the metabolism, biological effects and toxicity of the use of resveratrol to treat pathological and metabolic diseases in humans [15]. Although resveratrol appears to be well absorbed (~75%), primarily through transepithelial diffusion, by humans when taken orally, its bioavailability is relatively low (<1%) due to its rapid metabolism in the intestine and liver involving glucuronidated and sulfated compounds to generate key metabolites, *trans*-resveratrol-3-*O*-glucoronide and *trans*-resveratrol-resveratrol-3-sulfate, respectively, and elimination of these metabolites [16,17]. Preliminary studies found that the administration of single oral doses of 25 mg of *trans*-resveratrol to healthy volunteers resulted in peak/maximal blood concentrations (*C*_max_) of total resveratrol (*i.e.*, *trans*-resveratrol plus its metabolites) around 60 minutes later, at about 1.8-2 μmoles/L, depending on whether resveratrol was administered in wine, vegetable juice, or grape juice [16,17]. The serum levels of unmetabolized resveratrol peak in the sub- to low-micromolar range (<3 μmoles/L) within minutes of oral drug administration, and decrease rapidly thereafter, indicating low systemic availability due to rapid absorption and metabolism [16,17]. Consequently, a patented formulation of micronized resveratrol (SRT501), believed to enhance bioavailability by increasing surface area for intestinal absorption was tested in patients with stage-IV colorectal cancer and hepatic metastasis scheduled to undergo hepatectomy [18]. Administration of 5 g of SRT501 formulation in these patients resulted in 3.6-fold increase in *C*_max_, from 538 ng/mL (2.36 μmoles/L) to 1942 ng/mL (8.51 μmoles/L) in almost double the time, from 1.5 h to 2.8 h after intake, indicating a much slower absorption of the micronized form of resveratrol [18,19]. However, delay in the absorption due to disease pathology in these patients cannot be ruled out. Of note, this is in contrast to the markedly high doses of resveratrol (up to 200 μmoles/L) that are used in *in vitro* experiments to elicit sustained biological effects raising substantial concern that the concentrations used *in vitro* and in animal models are not reasonably attainable *in vivo*.

Resveratrol bioavailability exhibits a high inter-individual variability independent of age and gender [20]. Similarly, Bode *et al.* [21] concluded that resveratrol metabolism by human gut microbiota shows pronounced inter-individual differences based on the investigation of health-related effects of this stilbene. Up to nearly 20 resveratrol-derived metabolites have been described in plasma, urine and some tissues according to different studies in animals [22] and humans [23,24]. Among these metabolites, there are *trans*- and/or *cis*- forms of mono- and diglucuronides, mono- and disulfates and sulfoglucuronides from parent resveratrol, as well as equivalent conjugations of the microbiota-derived metabolite dihydro-resveratrol (DHRES). The activity of any specific resveratrol circulating metabolite is still unclear. Although DHRES has been reported to be the most abundant resveratrol-derived metabolite in colon from rats [25] and pigs [22], little is known of the biological relevance of DHRES.

The possible effect of the food matrix on resveratrol bioavailability has also been investigated. The main outcome has been that resveratrol-containing liquid formulations (either added or endogenous resveratrol) such as grape juice, vegetable juice, wine (white, red, sparkling) show similar absorption figures; high-fat foods delayed absorption; and neither alcohol nor the flavonol quercetin affected resveratrol bioavailability [26].

The basic parameters associated with any drug activity are its maximal plasma concentration (*C*_max_), time to *C*_max_, half-life and exposure measured by area under curve. These outputs can be dose and time dependent. Considering the fact that rapid absorption and metabolism of resveratrol is often believed to be a potential limitation of its activity and biological effects, it remains to be seen whether maximizing any of these parameters is important for the clinical efficacy of resveratrol. Nevertheless, with continued efforts in this direction numerous strategies have been described to improve the bioavailability of resveratrol, including use of micronized powders (e.g., SRT501), combining resveratrol with other polyphenols targeted by key enzymes, such as cytochrome P450 (CYP), UDP-glucuronosyltransferase (UGT), and sulfotransferase (SULT) which conjugate resveratrol, use of resveratrol precursors or pro-drugs to maximize the bioavailability of free *trans*-resveratrol, use of alternative delivery routes beyond traditional oral administration or controlled release devices (e.g., implants) to bypass gastrointestinal and hepatic metabolism, and nanotechnological formulations that have been reviewed elsewhere [27,28,29]. In addition, use of methylated polyphenol analogs (e.g., pterostilbene [3,5-dimethoxy-4′-hydroxy-trans-stilbene], the dimethylether analogue of resveratrol) may also help overcome these limitations to achieve pharmacologic efficacy [30].

## 4. Mode of Action

Resveratrol, originally isolated by Takaoka in the 1940s from hellebore roots (*Veratrum grandiflorum* O. Loes), and later by Nonomura in the 1960s from the Japanese knotweed *Polygonum* *cuspidatum*, had historically been used for remedial purposes in the traditional oriental medicine against various diseases including gonorrhea, athlete’s foot, suppurative dermatitis, and fungal skin diseases for its anti-microbial activity [31]. It was only in the 1990s that this compound was detected in wine and cardioprotective property attributed [32]. However, it was only after the *Science* publication by Jang *et al.* [33], demonstrating chemo-preventive activity of resveratrol and subsequently from reports that it activates sirtuin deacetylases, which extends the life-span in yeast [34], that studies on the effects and properties of this compound started accumulating exponentially. Since then, many studies have been published with varying degrees of evidence that resveratrol could exert a plethora of health benefits in a wide range of diseases, including cancer, cardiovascular and neurodegenerative diseases through different mechanisms of action.

A critical first step to delineate the mechanisms of drug action is to determine whether resveratrol mediates its effect by cell surface receptors or intracellular targets. Resveratrol is a hydrophobic compound, and has been demonstrated to be taken up by intestinal epithelium cells, hepatocytes and breast tumor cell lines [35,36,37]. Although both intra- and extracellular resveratrol targets have been proposed, a direct-binding partner has yet to be convincingly established. The vast majority of studies dealing with the biological activity of resveratrol have been primarily investigated *in vitro*, to a lesser extent in animal models and relatively few human clinical trials. Resveratrol exists in both *cis* and *trans* forms and *trans* form is believed to be more stable. Resveratrol is shown to be rapidly absorbed, both in *in vivo* human studies and *in vitro* cell culture studies, and is conjugated to form resveratrol glucoronide and resveratrol sulfate [38]. Resveratrol is known as an anti-aging, anti-cancer, anti-diabetic, neuroprotective and cardioprotective agent that acts by modulating various physiological processes, including oxidative stress, cell proliferation, apoptosis, inflammation, metastasis and angiogenesis, as shown in Table 2. Based on the current literature, some of the main biological activity of resveratrol, its effects and plausible mechanism(s) of action as demonstrated in different *in vitro* and *in vivo* conditions related to cancer [39,40,41], cardiovascular [42,43,44,45,46,47] and neurodegenerative [48,49] diseases have been outlined in Table 2. The precise molecular mechanism(s) behind the pleiotropic beneficial effects of resveratrol is still unclear and remains controversial. However, the current evidence does suggest that resveratrol primarily acts via direct and indirect activation of the histone deacetylase silent mating type information regulation 2 (Sir2) homolog 1 (SIRT1) both *in vitro* and *in vivo* [50,51]. An initial study by Howitz *et al.* demonstrated that resveratrol stimulated the activity of the NAD^+^-dependent deacetylase SIRT1, the mammalian ortholog of Sir2 of the sirtuin family in yeast, reported to be associated with longevity [52]. However, subsequent studies added uncertainty to the conclusion that resveratrol is a direct stimulator of SIRT1 function [53] and in fact suggested that resveratrol stimulates the AMP-activated protein kinase (AMPK) pathway by directly inhibiting cAMP-degrading phosphodiesterases [54]. To establish clear biochemical and genetic link between SIRT1 and resveratrol, Price *et al.* demonstrated that the specific physiological and beneficial effects of resveratrol are lost in the absence of a functional SIRT1 in SIRT1-knockout mice [51]. The study showed that at low-to-moderate doses of resveratrol SIRT1 is necessary for AMPK activation and NAD^+^ increase and that SIRT1 acts upstream of AMPK [51]. Interestingly, at higher doses these effects are SIRT1-independent [51], indicating that the mechanism is dose-dependent. SIRT1 activation plays a critical role in signaling pathways related to apoptosis, autophagy, insulin sensitivity and benefits of caloric restriction diet [51,55]. It is extremely noteworthy that many of the reported biological activities of resveratrol were observed in cells cultured in the presence of resveratrol at much higher concentrations than those likely to be achieved in humans *in vivo*. Nonetheless, studies have demonstrated that even low concentrations of resveratrol are beneficial [56,57]. Resveratrol has been shown to increase endothelial nitric oxide synthase activity (eNOS) at 0.1–1 μmoles/L concentration in a human endothelial cell line after only two minutes of incubation [57], and increase AMPK activation, via SIRT1, in human vascular smooth muscle cells at a concentration of 3 μmoles/L [56]. The myriad of pathways and molecules that are altered in response to resveratrol treatment have been reviewed elsewhere [13]. In addition, a recent publication by Tomé-Carneiro *et al.* [58] has thoroughly and very elegantly reviewed the most relevant preclinical studies (*in vitro* and animal models) and human intervention studies published in the most recent decades, highlighting the different biological significance and plausible mechanisms of action of resveratrol.

## 5. Safety

### 5.1. Adverse Effects

Studies in animal model have shown no apparent adverse effects on administration of high doses of (*trans*-) resveratrol [59]. However, studies in humans are relatively few and are reviewed elsewhere [15,58]. Short-term studies have reported mild-to-moderate gastrointestinal side effects, including nausea, abdominal pain, flatulence, and diarrhea at concentrations of about 2.5 to 5 g doses [19,26]. Other adverse effects reported at 5 g of micronized resveratrol (SRT501) include chills, lethargy, rash, skin irritation and vascular flushing that resolved spontaneously without sequelae [18]. Post-operative peritonitis, liver failure and death deemed unrelated to the study drug (SRT501) was reported in a single patient [18]. However, evaluation of resveratrol safety for long-term usage needs to be performed. The safety of resveratrol-containing supplements during pregnancy and lactation has not been established; theoretically, pregnant women should avoid consuming wine as a source of resveratrol. Moreover, women with a history of estrogen-sensitive cancers (breast, ovarian, and uterine) should avoid resveratrol supplements until more is known about the estrogenic activity of resveratrol in humans [60].

### 5.2. Drug Interactions

Resveratrol can inhibit human platelet aggregation *in vitro* [32]. Therefore, theoretically, high intakes of resveratrol (*i.e.*, from supplements) could increase the risk of bruising and bleeding when taken with anticoagulant drugs, such as warfarin and heparin; antiplatelet drugs, such as clopidogrel and dipyridamole; and non-steroidal anti-inflammatory drugs, including aspirin, ibuprofen, diclofenac, naproxen, and others.

### 5.3. Drugs Metabolized by Cytochrome P450

Cytochrome P450 3A4 (CYP3A4) is one of the most abundant CYP isoform enzymes, which catalyzes metabolism of most of the drugs available in the market affecting clearance and toxicity of the drug. Resveratrol has been shown to inhibit CYP3A4 activity *in vitro* [61] and in healthy volunteers [62]. In a very recent study, α-Viniferin isolated from *Caragana* *chamlagu*, a trimer of resveratrol, strongly inhibited CYP2C19-mediated omeprazole 5-hydroxylation and CYP3A4-catalyzed midazolam 1-hydroxylation with IC50 values of 0.93 and 1.2 μM, respectively [63]. These two CYPs were inhibited in a dose-dependent but time-independent manner. Other CYP enzymes (e.g., CYP1A2, CYP2D6 and CYP2C9) may also be inhibited by resveratrol [62].

## 6. Resveratrol and Eye Diseases

Many studies have investigated the effects of resveratrol within the eye and its related disorders. Similar to the effects observed in other diseases, such as cancer, heart and neurodegenerative diseases (discussed above) the major biological actions on the eye include: anti-oxidative, anti-apoptotic, anti-tumorogenic, anti-inflammatory, anti-angiogenic and vasodilator. Studies investigating these effects and their potential role in the progression of eye diseases including glaucoma, cataract, age-related macular degeneration and diabetic retinopathy have been described in this section.

### 6.1. Glaucoma

Glaucomas are a group of progressive optic neuropathies characterized pathologically by apoptosis of retinal ganglion cells (RGCs) and resulting changes in the optic nerve head. Loss of RGCs, at least in part, is related to the level of intraocular pressure (IOP) as a consequence of blockage of aqueous outflow pathway through the trabecular meshwork. There may also be mitochondrial dysfunction in the RGCs, as a result of high levels of energy demand that may be difficult to meet during periods of intraocular pressure-induced metabolic stress. Although reduction of IOP is the only proven effective method to treat the disease, ocular hypotensive drops, laser trabeculoplasty and surgery may also be used to slow disease progression. Pathways involving oxidative stress, inflammation, mitochondrial dysfunction, glial cell dysfunction, and activation of apoptosis and cell survival are active areas of research to identify new potential drug targets [1,64].

Luna *et al.* investigated the therapeutic effects of administration of the dietary supplement resveratrol on the expression of glaucoma markers in trabecular meshwork cells subjected to chronic oxidative stress [65]. The study demonstrated that resveratrol effectively decreased the production of intracellular ROS (iROS) and inflammatory markers such as interleukin-1 alpha (IL-1α), interleukin (IL)-6, IL-8 and endothelial-leukocyte adhesion molecule-1 (ELAM-1). In addition, the expression of the cellular senescence marker sa-β-galactosidase (sa-β-gal), typically induced by chronic oxidative stress, lipofuscin, the end product of lipid peroxidation, and other carbonylated proteins, was also found to be reduced by resveratrol treatment. The study concluded that resveratrol could potentially have a significant anti-apoptotic role in preventing damage to the trabecular meshwork cells consistently observed in POAG patients without having a detrimental effect on cell proliferation. In addition, the study also inferred that this effect was not as a result of decreased protein degradation, since proteosomal activity was not affected by resveratrol [65]. The study provided evidence for anti-oxidative and anti-apoptotic role of resveratrol in trabecular meshwork cells.

There is progressive loss of retinal ganglion cells (RGCs) in glaucoma and therefore neuroprotection of RGCs may have an impact on management of glaucoma. A recent study by Prihan and colleagues [66] investigated the effect of administration of riluzole and resveratrol alone or in combination on the survival of RGCs in experimental rat model of glaucoma. Riluzole is a neuroprotective FDA-approved drug treatment due to life-prolonging effects in amyotrophic lateral sclerosis (ALS). However, its exact mechanism of neuroprotection is not known yet. The study included an early (starting with glaucoma induction) and late (three weeks after glaucoma induction) riluzole-treated and resveratrol-treated groups, in addition to an early riluzole and resveratrol combination group along with glaucoma (vehicle control) and control (no medication) groups followed through a period of six weeks. The study demonstrated that both riluzole and resveratrol therapy offered significant neuroprotective effect on RGC survival in the experimental model of glaucoma. Furthermore, therapy initiated in the early phase was found to be more effective than late treatment as indicated by RGC density count for both the agents. In addition, the combined use of both these agents was associated with significantly higher survival rate of RGCs as compared to either riluzole or resveratrol monotherapy. The precise mechanism by which riluzole and resveratrol produce neuroprotection in glaucoma is not clear, however, based on the pronounced effect observed in combined-therapy, it was hypothesized that the neuroprotective ability may be as result of distinct mechanisms of action of riluzole and resveratrol, the capacity of acting at different stages of glaucomatous injury or the synergistic effects of the two agents on excitotoxicity and neuromodulation. Despite certain limitations such as short-term study, lack of axonal counts, functional data (e.g., electroretinogram) and time and dose-dependent experiments, the study highlighted a promising role for resveratrol in glaucoma treatment. Another recent study by Lindsey *et al.* [67] investigated the effect of long-term dietary supplementation with resveratrol on loss of dendrite RGC following optic nerve injury and the resolution of unfolded protein response in young-adult Thy1-yellow fluorescent protein (YFP) and C57BL/6 mice. Following three weeks after optic nerve crush, mice receiving resveratrol showed reversal of cytoplasmic binding immunoglobulin protein (BiP) suppression (that was observed in ganglion cell layer neurons of mice on control diet), moderate increase in nuclear C/EBP homologous protein (CHOP), and reduction in nuclear X-box binding protein-1 (XBP-1) to the same levels as in control diet uncrushed optic nerve eyes. These results substantiated the findings of previous study [66] emphasizing the neuroprotective role of resveratrol-mediated protection of RGC dendrites. In addition, the study showed that protection of dendrites by resveratrol following optic nerve injury involves alterations in long-term expression of BiP, CHOP and XBP-1 and that it varies with RGC types [67].

Steroid-induced ocular hypertension (SIOH) and glaucoma is associated with increased extracellular deposition in trabecular meshwork and are treated in the same way. Two studies were recently published by Razali and group, evaluating the use of topical trans-resveratrol-induced intraocular pressure (IOP) reduction in rats with SIOH [68]. The first study reported a significant reduction in IOP in SIOH rats and attributed this lowering to activation of adenosine A1 receptors, which may lead to increased activity of matrix metalloproteinase (MMP)-2. The effect was abolished by pre-treatment with A1 antagonist but not in the presence of A_2_A and A_3_ AR antagonists [68]. The study evaluated the effect of repeated topical application of trans-resveratrol for 21 days in SIOH rats. The study concluded that repeated usage of topical application of trans-resveratrol produced sustained lowering of IOP which was associated with increased levels of aqueous MMP-2, normalization of TM morphology as indicated by significantly reduced TM thickness and increased number of TM cells, improved retinal morphology and restoration of retinal redox status [68]. These studies demonstrated oculo-hypotensive effect of topical trans-resveratrol in both normotensive and oculo-hypertensive rats involving agonistic action at A_1_ AR, at least in part. Treatment with topical steroids is known to increase IOP by increasing the resistance in the aqueous humor outflow pathways, which is also observed in POAG eyes [69]. Therefore, topical trans-resveratrol-induced IOP reduction is important not only in SIOH but also in POAG eyes. These findings, however, need to be confirmed in human studies. Moreover, further investigations involving ocular pharmacokinetics of topically applied trans-resveratrol and post-receptor signaling mechanisms that contribute to its IOP lowering effect will provide additional insights into the potential use of trans-resveratrol as an IOP reducing agent in SIOH and glaucoma. In addition, Liu *et al.* has shown that resveratrol may be able to protect against retinal ischemia/reperfusion injury induced by high intraocular pressure (namely 120 mmHg for 60 min) by downregulation of *MMP-9* and *iNOS*, and upregulation of *HO-1* [70].

The role of mitochondrial dysfunction in the pathogenesis of glaucomatous neurodegeneration has been of considerable interest in the recent past. Chen and colleagues have evaluated the role of resveratrol-triggered mitochondrial biogenesis for preventing apoptosis in a retinal ganglion cell line RGC-5 [70]. Serum deprivation induced RGC-5 cell apoptosis by impairing mitochondrial function. Treatment with resveratrol considerably retarded the apoptotic progress, maintaining the normal mitochondrial membrane potential, together with decreased levels of both total and cleaved caspase-3, and inhibition of the release of cytochrome c, which subsequently enhanced cell survival. Decreased level of expression of cleaved caspase-3 (a marker of apoptotic signaling events) suggest that resveratrol could shield RGC-5 cells from serum deprivation-elicited apoptosis by maintaining their mitochondrial integrity. Moreover, resveratrol stimulated mitochondrial biogenesis by increasing the absolute quantity of mitochondria as well as their DNA copies. Treatment with resveratrol also promoted the protein expression of SIRT1, but not peroxisome proliferator activated receptor-c co-activator 1 alpha (PGC-1α). Instead resveratrol facilitated PGC-1α translocation from the cytoplasm to the nucleus and up-regulated nuclear-encoded respiratory complex protein (NRF1) and mitochondrial transcription factor A (TFAM). PGC-1α is the predominant coactivator in the regulation of mitochondrial biogenesis controlling transcription of nuclear-encoded respiratory complex proteins (NRF1) and mitochondrial transcription factorA (TFAM) [71,72]. SIRT1, member of sirtuin family, is a mammalian homolog of yeast Sir2, a protein responsible for transcription silencing that has been linked to lifespan extension [73]. SIRT1-dependent de-acetylation and subsequent activation of PGC-1α are significant metabolic events to meet the high energy requirement of cells [72]. The study provided evidence that resveratrol treatment could potentially attenuate serum deprivation-elicited RGC-5 cell death via the SIRT1-dependent PGC-1α subcellular translocation, thereby raising the possibility of mitigating glaucomatous retinopathy by promoting mitochondrial biogenesis [70]. It would very interesting to elucidate this mechanism *in vivo* in experimental glaucoma models.

### 6.2. Age-Related Macular Degeneration

Age-related macular degeneration (AMD) is closely associated with choroid-Bruch’s membrane (BM)-retinal pigment epithelium (RPE)-neuroretina (NR) component in the posterior part of the eye in the aging population [74]. “Dry” AMD is characterized by accumulation of drusen (lipofuscin) around RPE with some degree of RPE degeneration [74]. “Wet” AMD is characterized by the presence of choroidal neovascularization (CNV) in which newly formed blood vessels radiate outward from the choroid, penetrate through BM, and proliferating into the RPE or subretinal space in NR [74]. There is growing evidence to suggest a role for retinal pigment epithelial (RPE) cell damage and death, caused by different mechanisms including inflammation and oxidative stress in AMD causing photoreceptor death and loss of vision [75,76]. There are compelling evidence to indicate association of vascular endothelial growth factors (VEGF) to CNV processes in AMD, so anti-VEGF therapies are the main focus for the management of AMD [77]. In addition, several prospective, retrospective, randomized controlled trials and cross-sectional studies have been conducted to find potential nutrients and other supplements that can delay or prevent AMD pathologies [78].

Resveratrol prevents oxidative stress-induced and sodium iodate-induced apoptosis of human RPE cells *in vitro* [79]. Moreover, proliferation of RPE cells has been shown to be reduced via inhibition of extracellular signal-regulated protein kinases one and two (ERK1/2) and mitogen activated protein kinase (MAPK) signaling cascade [79,80]. Resveratrol has also been shown to protect RPE cells from autoimmune antibodies-induced apoptosis, *in vitro*, which is highly relevant in autoimmune-associated retinopathies [81]. It was suggested that resveratrol down-regulated pro-apoptotic Bcl-2-associated X protein (BAX) within the mitochondrion and cytoplasm of antibody-treated cells by inhibiting intracellular increase of Ca^2+^ ion concentration; and up-regulated SIRT1 and Ku70 anti-apoptotic proteins leading to suppression of BAX translocation within the mitochondria [81,82]. In addition, Kubota *et al.* reported a protective effect of resveratrol against light-induced retinal degeneration, a model used to investigate visual cell apoptosis, by reversing the activation of retinal activating protein-1 (AP-1) and inhibition of SIRT1 activation up-regulated by light exposure [83]. AP-1 regulates cell proliferation and apoptosis; and retinal extracts of resveratrol treated mice were found to down-regulate c-fos expression, which in turn suppressed AP-1 activation preventing the death of photoreceptor cells [83]. The anti-apoptotic activity of resveratrol seems to be an important contributing factor in the prevention of disease of a neurodegenerative nature, such as AMD.

Unlike many *in vitro* studies, there is a limited evidence for the anti-oxidative effect of resveratrol *in vivo* that may be relevant to AMD. Resveratrol was reported to exhibit a dose-dependent protective effect against hydrogen peroxide-induced cytoxicity in human retinal D407 RPE cells by increasing superoxide dismutase, glutathione peroxidase, and catalase activities that inhibited the levels of intracellular ROS [84]. A similar effect was also reported by Zheng *et al.* in human lens epithelial cells [85]. Although limited, these studies emphasize the importance of reducing the amount of oxidation that occurs in the retina of AMD patients and suggests that the anti-oxidative effect of resveratrol supplementation may be beneficial in preventing RPE degeneration induced by oxidative stress in these patients.

Pathogenic late stages of AMD are often characterized by choroidal angiogenesis. The anti-inflammatory and anti-oxidative properties of resveratrol are believed to reduce the incidence of CNV. Nagineni and colleagues investigated the inhibitory actions of resveratrol on inflammatory cytokine, transforming growth factor-beta (TGF-β) and hypoxia induced VEGF secretion by human RPE cells to demonstrate utility of resveratrol as nutraceutical supplement in controlling CNV processes in AMD [86]. The study showed that resveratrol suppressed inflammatory cytokine mix, TGF-β and hypoxia enhanced VEGF-A and VEGF-C secretion by human RPE without influencing anti-angiogenic endostatin and pigment epithelial derived factor secretion. In addition, resveratrol down-regulated expression of nuclear factor-kappaB (NFκB) and hypoxia-induced factor (HIF)-1α transcription factors, thereby inhibiting VEGF secretion. SIRT1, a major modulator of resveratrol, represses HIF-1α signaling by deacetylation leading to reduced VEGF-A secretion [87]. Resveratrol has also been shown to act via activation of eukaryotic elongation factor-2 (eEF2) kinase and inhibit VEGF secretion, endothelial cell proliferation and migration by a novel SIRT1 independent pathway to prevent pathologically aberrant injury-induced angiogenesis [88]. A report on resveratrol-based nutritional supplementation on three octogenarians with AMD observed a short-term effect similar to that found with anti-VEGF treatment including anatomical restoration of retinal structure, improved RPE function, and a suggested improved choroidal blood flow [89]. In another recent study, Richer *et al.* reported broad bilateral improvements in ocular structure and function in three patients with AMD over a long-term follow-up of two to three years suggesting its efficacy in AMD [90].

Resveratrol positively affects mitochondrial biogenesis and has been shown to protect RPE cells against acrolein-induced oxidative cytotoxicity by increasing mitochondrial bioenergetics [91].

### 6.3. Diabetic Retinopathy

Diabetic retinopathy (DR) is a diabetes-induced microangiopathy affecting the retinal vasculature due to poor metabolic control of circulating blood glucose levels characterized by increased inflammatory response, ischemia, progressive RPE cell degeneration leading to malfunction of blood-retinal barrier and loss of vision [92]. Supplementation of resveratrol in diabetic rats has demonstrated significant alleviation of hyperglycemia, weight loss, enhancement of oxidative markers, superoxide dismutase activity, and suppression of eNOS activity in the blood and retina [93]. Treatment with resveratrol has been shown to suppress diabetic changes such as increased vessel leakage, pericyte loss and VEGF protein levels in the retinas of mice induced with diabetes [94]. Li and colleagues have shown that resveratrol can inhibit endoplasmic reticulum stress, which significantly contributes to retinal vascular degeneration [95]. In another study, Losso *et al.* investigated the ability of resveratrol to inhibit RPE cell inflammation caused by hyperglycemia *in vitro* [96]. The study reported that cells treated with resveratrol significantly inhibited the accumulation of VEGF, TGF-β1, cyclo-oxygenase-2 (COX-2), IL-6 and IL-8 in a dose-dependent manner. In addition, the activity of protein kinase C-beta (PKCβ), was also significantly reduced in the presence of resveratrol. PKCβ is known to up-regulate VEGF activity in hypoxic conditions further contributing to the degradation of blood-retina barrier [96].

### 6.4. Other Eye Related Diseases

Few studies have also indicated the potential preventative role of resveratrol in other eye related diseases. Doganay *et al.* demonstrated a role for resveratrol in rat against selenite-induced cataract formation by stimulating an increase in the levels of reduced glutathione (GSH) and reduced concentrations of malondialdehyde (MDA), a marker of lipid peroxidation, in rat lenses and erythrocytes [97]. Administration of resveratrol to an endotoxin-induced uveitis (EIU) mouse model of ocular inflammation showed significant decline in oxidative damage (8-hydroxy-2′-deoxyguanosine generation) and suppression of NF-kB p65 translocation (NF-kB activation), leading to ocular anti-inflammatory effect (reduced levels of MCP-1 and ICAM-1) in the retina and the RPE-choroid [98]. Resveratrol treatment has also been demonstrated to cause tumor cell death and regression. Resveratrol was found to inhibit uveal melanoma tumor growth via early mitochondrial dysfunction and caspase-3 activation [99]. Similarly, Sareen and colleagues showed that resveratrol inhibited growth of retinoblastoma cell line in a time- and dose-dependent manner by promoting cell cycle S-phase arrest and apoptosis [100]. In another study, Kim *et al.* have demonstrated effectiveness of resveratrol as a potential treatment in *in vivo* and *in vitro* rat models of oxygen-induced retinopathy via nitric oxide- (NO) mediated pathways, suggesting a protective role in retinopathy of prematurity (ROP) in prematurely born infants [101]. Resveratrol supplementation has shown no effect on corneal neovascularization on experimental corneal alkali burn rabbit model [102].

## 7. Current Limitations and Future Perspective

The numerous *in vitro* and animal experimental studies conducted so far have provided evidence for the biological effects of resveratrol on several pathways including oxidative stress, inflammation, mitochondrial dysfunction, apoptosis, pro-survival or angiogenesis that are implicated in the pathogenesis of various eye diseases discussed above. These studies have also suggested many possible direct or indirect molecular targets and mechanisms of action mediating them.

Although a wide variety of pharmacologic activities of resveratrol has been documented, it is still unclear how the same drug causes different or even opposite effects in different cell types and in different diseases. For example, resveratrol causes cell death in tumor cells but is effective as an anti-apoptotic or protective agent in nerve cells [103]. Similarly, resveratrol increases mitochondrial health in muscle cells, whereas it induces mitochondria-mediated apoptosis in tumor cells [104]. In addition, many effects and mechanisms proposed to be regulated by resveratrol are not yet confirmed in humans. Further research using the human clinical studies and randomized, double-blinded placebo-controlled experimental protocols are needed to confirm these and other possible effects and mechanisms in order to determine its efficacy and full potential as a component of ocular nutritional supplements. However, it would be a challenge to conduct a perfect human clinical trial perhaps, because the pharmaceutical industry may not be interested in investing funds on human research since resveratrol is readily-available for everyone as a dietary supplement and the available general beneficial findings of resveratrol are promptly used by the nutritional-based companies who do not perform any type of research. This has contributed to an endless loop in resveratrol research thus far.

The poor bioavailability of resveratrol is another major drawback for this molecule and even a recurrent criticism used by some physicians or pharmacologists, *i.e.*, “resveratrol cannot exert benefits because it is rapidly metabolized and its presence in the bloodstream is negligible to justify any effect”. Goldberg *et al.* [16], in 2003, pointed out that the huge output of studies reporting *in vitro* effects of free resveratrol could not be relevant as it was absorbed as conjugates. Azorín-Ortuño *et al.* [22] coined the expression “Resveratrol Paradox” to illustrate the wide variety of activity exerted by resveratrol despite its low bioavailability. Information about the bioavailability of resveratrol in humans is very important since most of the experimental research conducted to date has been “preclinical,” *i.e.*, *in vitro*, exposing cells to high doses of resveratrol (and short follow-ups to maximize effects in a short time), which are up to 100 times higher than peak plasma concentrations observed in humans, and in animal models given very high (non-dietary) doses of resveratrol [58]. At present, very little is known about the biological activity of resveratrol metabolites. The specific biological activity of circulating metabolites has been scarcely approached due to the lack of suitable standards [105]. The “actual metabolite” responsible (if any) for the effects exerted following resveratrol intake is still obscure. The objective of using high doses of resveratrol with a pharmacological use is still unclear. The evaluation of effects of low doses of resveratrol supplementation is challenging because it requires long-term trials to exert quantifiable effects. The specific dose of resveratrol needed to maximize effects without safety concerns is not known yet. Few studies have investigated different concentration of resveratrol in an effort to define lethal dose [99,100]. These approaches will enable identification of the possible “*actual metabolite*” responsible for the diverse effects exerted by resveratrol and provide knowledge on the optimal effective concentrations of resveratrol that could be beneficial and yet safe to the subject.

Based on current literature, it would seem that resveratrol may have the potential to ameliorate secondary conditions even as it is used to treat a primary disease via the plausible biological mechanisms demonstrated using *in vitro* experiments and animal models of several ocular diseases. However, data in human research is lacking and it is essential to carry out comprehensive randomized clinical trials on both the preventive potential, efficacy and safety of resveratrol in different diseases before such claims can be validated. There are a number of ongoing clinical trials dealing with resveratrol [106] that will provide more insights about its effect(s) on human health in the near future.

## Figures and Tables

**Table 1:** Trans-resveratrol concentration estimates in selected sources [11].

Source	Trans-Resveratrol Concentrations
Red wines	0.1–14.3 µg/mL
White wines	<0.1–2.1 µg/mL
Cranberry raw juice	~0.2 µg/mL
Grapes	0.16–3.54 µg/g
Blueberries	Up to ~0.032 µg/g
Bilberries	Up to ~0.016 µg/g
Peanuts	0.02–1.92 µg/g
Boiled peanuts	5.1 µg/g
100% Natural peanut butters	0.65 µg/g (average)
*Polygonum cuspidatum* herb	524 µg/g

**Table 2:** Some of the main biological activity, their effects and plausible mechanisms of action of resveratrol as demonstrated in different *in vitro* and *in vivo* conditions.

Biological Activity	Effect	Plausible Mechanism(s)	Reference
Anti-proliferative	Induction of apoptosis and/or cell cycle arrest	Caspase activation, inhibition of Bcl-2 proteins, PI3K/Akt/mTOR pathway, transcription factors; modulation of cyclins and cyclin-dependent kinases balance	[39,40]
Anti-angiogenesis	Inhibition of tumor growth, cell migration, invasion and metastasis	Decreases expression of leukotriene B4 and matrix metalloproteinases (particularly, MMP9)	[41]
Anti-oxidative	Inhibition of reactive oxygen species (ROS)	Induction of superoxide dismutase, catalase, glutathione peroxidase-1	[42]
Endothelial	Increased activity and/or expression of endothelial nitric oxide synthase (eNOS)	via 5′-adenosine monophosphate activated protein kinase (AMPK) or extracellular signal-regulated kinase 1/2 (ERK 1/2-mediated phosphorylation; activating histone/protein deacetylase silent information regulator 2/sirutin 1 (SIRT1)	[43,44]
Anti-inflammatory	Attenuated DNA damage and upregulation of IL-6, TNF-a	via SIRT1 activation	[45]
	Reduces expression of adhesion molecules (ICAM-1 and VCAM-1)	Inhibition of p38 MAPK signaling pathway	[46]
Anti-platelet	Inhibition of platelet activation and aggregation	Inhibition of p38 MAPK pathway and activation of NO/cyclic guanosine monophosphate causing inhibition of phospholipase C and/or protein kinase C activation	[47]
Pro-proliferative and neurogenesis	Inhibition of apoptosis, inflammation and oxidative stress; enhances neuronal cell survival and improves cognitive behavior	Regulation of HO-1 and peroxisome proliferator activated receptor gamma coactivator 1 alpha (PGC-1a)	[48,49]
